# An improved strategy to analyse strigolactones in complex sample matrices using UHPLC–MS/MS

**DOI:** 10.1186/s13007-020-00669-3

**Published:** 2020-09-17

**Authors:** Kristýna Floková, Mahdere Shimels, Beatriz Andreo Jimenez, Nicoletta Bardaro, Miroslav Strnad, Ondřej Novák, Harro J. Bouwmeester

**Affiliations:** 1 Plant Hormone Biology Group, Swammerdam Institute for Life Sciences, University of Amsterdam, Science Park 904, 1098 XH Amsterdam, The Netherlands; 2 Laboratory of Growth Regulators, Institute of Experimental Botany, The Czech Academy of Sciences, and Faculty of Science, Palacký University, Šlechtitelů 27, 783 71 Olomouc, Czech Republic; 3 Laboratory of Plant Physiology, Wageningen University, Droevendaalsesteeg 1, 6708 PB Wageningen, The Netherlands; 4 Department of Microbial Ecology, Netherlands Institute of Ecology, Droevendaalsesteeg 10, 6708 PB Wageningen, The Netherlands; 5 Biointeractions and Plant Health, Wageningen University, Droevendaalsesteeg 1, 6708 PB Wageningen, The Netherlands; 6 Department of Plant, Soil and Food Science, Section of Genetics and Plant Breeding, University of Bari, Via Amendola 165/A, 70126 Bari, Italy

**Keywords:** Phytohormones, Strigolactones, UHPLC–MS/MS, Solid phase extraction (SPE), Quantitative analysis, Phosphate starvation

## Abstract

**Background:**

Strigolactones represent the most recently described group of plant hormones involved in many aspects of plant growth regulation. Simultaneously, root exuded strigolactones mediate rhizosphere signaling towards beneficial arbuscular mycorrhizal fungi, but also attract parasitic plants. The seed germination of parasitic plants induced by host strigolactones leads to serious agricultural problems worldwide. More insight in these signaling molecules is hampered by their extremely low concentrations in complex soil and plant tissue matrices, as well as their instability. So far, the combination of tailored isolation—that would replace current unspecific, time-consuming and labour-intensive processing of large samples—and a highly sensitive method for the simultaneous profiling of a broad spectrum of strigolactones has not been reported.

**Results:**

Depending on the sample matrix, two different strategies for the rapid extraction of the seven structurally similar strigolactones and highly efficient single-step pre-concentration on polymeric RP SPE sorbent were developed and validated. Compared to conventional methods, controlled temperature during the extraction and the addition of an organic modifier (acetonitrile, acetone) to the extraction solvent helped to tailor strigolactone isolation from low initial amounts of root tissue (150 mg fresh weight, FW) and root exudate (20 ml), which improved both strigolactone stability and sample purity. We have designed an efficient UHPLC separation with sensitive MS/MS detection for simultaneous analysis of seven natural strigolactones including their biosynthetic precursors—carlactone and carlactonoic acid. In combination with the optimized UHPLC–MS/MS method, attomolar detection limits were achieved. The new method allowed successful profiling of seven strigolactones in small exudate and root tissue samples of four different agriculturally important plant species—sorghum, rice, pea and tomato.

**Conclusion:**

The established method provides efficient strigolactone extraction with aqueous mixtures of less nucleophilic organic solvents from small root tissue and root exudate samples, in combination with rapid single-step pre-concentration. This method improves strigolactone stability and eliminates the co-extraction and signal of matrix-associated contaminants during the final UHPLC–MS/MS analysis with an electrospray interface, which dramatically increases the overall sensitivity of the analysis. We show that the method can be applied to a variety of plant species.

## Background

**Fig. 1:**
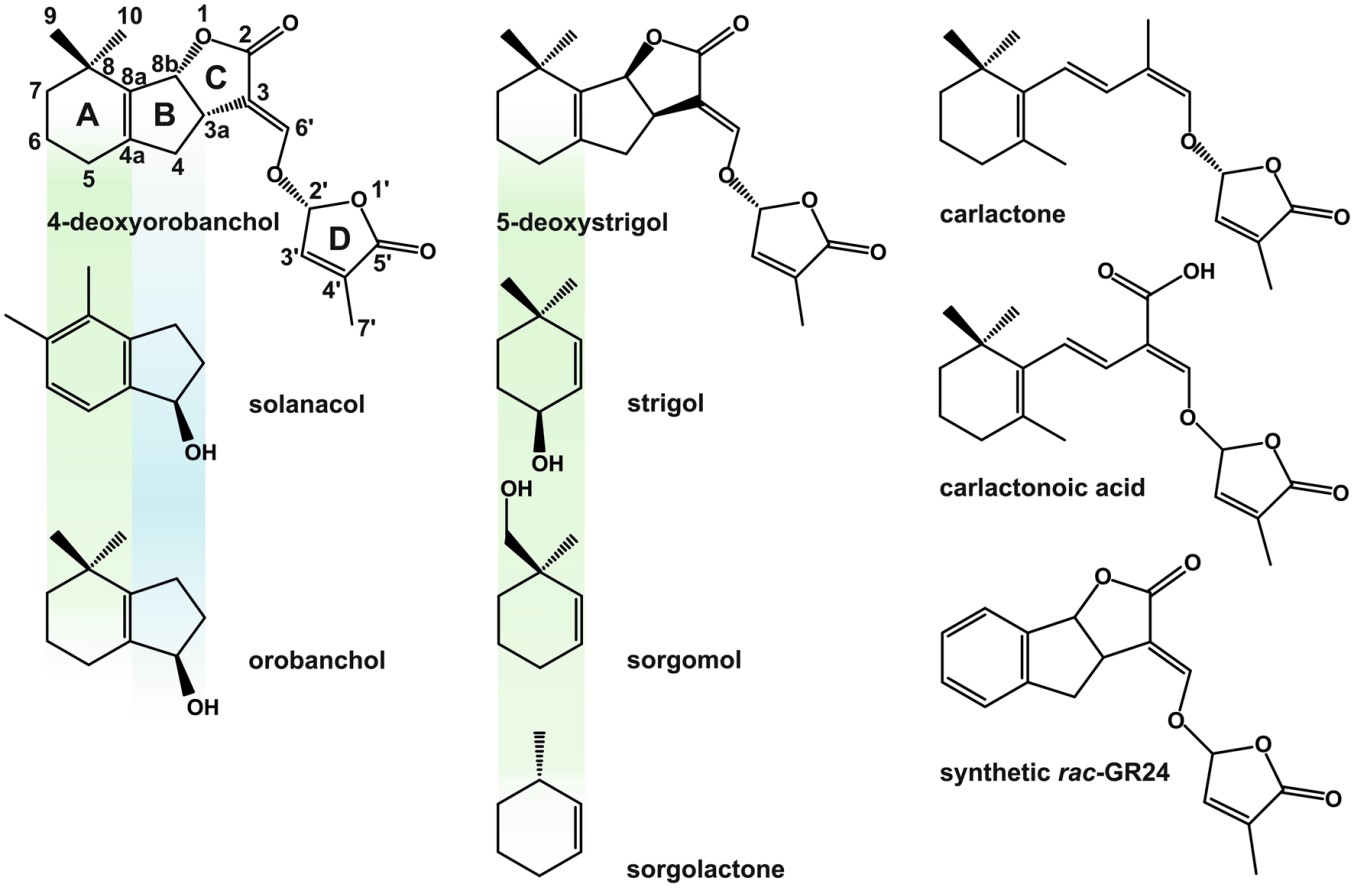
The structures of strigolactones. Structures of compounds investigated in the current study, varying in modifications of the A, B and C ring, including synthetic GR24 and non-canonical structures of strigolactone biosynthetic precursors—carlactone and carlactonoic acid. Only one enantiomer representative of racemic standard mixtures is shown

Strigolactones (SLs) are phytohormones that also have rhizosphere signaling activity. These root-exuded apocarotenoid lactones have been intensively studied during the past half-century as a germination factor of root parasitic *Striga*, *Orobanche* and *Phelipanche* spp. with strong negative effect on yield of agriculturally important crops [1]. In addition to this rhizosphere communication role, SLs control aspects of root and shoot architecture [2–4]. A broad range of mono- and dicotyledonous, and also lower plant species have been shown to produce a blend of SLs in different quantities and combinations of which the production is upregulated by phosphate and nitrogen deficiency [5–10]. The biological relevance of this structural diversity in the SLs remains unclear. A typical SL molecule, the canonical SLs, comprises four rings (A–D) of which the C–D part is highly conserved whereas the A and B rings show considerable variation due to modifications by different side groups (Fig. 1). This group contains the compounds such as strigol, solanacol, sorgomol, orobanchol, sorgolactone, 5-deoxystrigol (5-DS) and synthetic strigolactone mimic GR24 (Fig. 1). The non-canonical SLs have strongly modified A, B and/or C-rings and include biosynthetic precursors of the SLs-carlactone, carlactonoic acid (Fig. 1), and SLs such as heliolactone, avenaol, zealactone and zeapyranolactone [11–14]. In addition, the stereochemistry plays a significant role in SL biological activity [15]. The typical structural feature shared by all SLs is the enol-ether linkage on C-2′, connecting the butanolide D ring to the rest of the SL molecule (Fig. 1). This lactone-enol-lactone moiety is also responsible for the low stability of the SLs towards hydrolysis in aqueous solutions at pH ≥ 7.5 and in the presence of nucleophiles [16–19]. Detailed insight into the SL metabolome and tissue-specific analysis is complicated by this limited stability as well as extremely low concentrations of SLs in plant/soil matrices (fmol/g of root fresh weight; pmol/l of root exudate) [20, 21]. The strategy to overcome problematic extraction efficiency and loss of analyte during the sample preparation is, in general, to compensate this by using large initial sample size [20, 22]. Since the high-performance chromatography tandem mass spectrometry with the electrospray interface LC–ESI–MS/MS has become the method of choice in SL analysis, samples of appropriate purity are required in order to minimize the effect of co-eluting, interfering compounds during the ESI process. However, the broad polarity of SLs and the absence of ionizable moieties hinder the development of a tailor-made specific sample preparation procedure. In the early SL isolation and identification work, SLs were trapped from several liters of root exudates of hydroponically grown plants using charcoal or polystyrene-divinylbenzene based resins, and subsequently eluted by polar aprotic solvents such as acetone and ethyl acetate [5, 23, 24]. Liquid–liquid based extraction using ethyl acetate was applied to isolate strigol from root exudates of millet and root culture filtrate of the herbaceous plant *M. dauricum* [25, 26], as well as to quantify and characterize SLs in hydroponic cultures of other plant species such as cotton, red clover, sorghum and rice [21, 27–29]. Solid phase extraction (SPE) is the most commonly used method to concentrate and desalt root exudates based on the interaction of SLs with the long alkyl chains of C18 sorbents [6, 30], while root tissue is usually extracted with ethyl acetate and purified using silica to eliminate the presence of non-polar pigments and other lipids in the sample [20, 22, 28, 31]. More modern SPE sorbents consisting of polymeric materials are increasingly popular in plant hormone analysis. They have superior stability, combine different retention mechanisms simultaneously and, hence, facilitate sample preparation [32, 33]. Umehara et al. included the macroporous poly-divinylbenzene-co–*N*-vinylpyrrolidone—which combines hydrophilic and lipophilic retention characteristics—with “mixed-mode” anion exchanger in a multistep protocol to isolate 2′-*epi*-5-deoxystrigol, nowadays 4-deoxyorobanchol (4-DO), from roots and shoots of rice seedlings [34, 35]. The need for several concentration and purification steps make accurate, quantitative SL analysis inherently difficult. The addition of an appropriate internal standard in a known concentration before sample preparation is the best solution to correct for variation in the extraction recovery. Several laboratories have long standing experience in organic synthesis of SLs and (stable isotope-labelled) analogues [20, 31, 36]. However, the difficult and expensive synthesis complicates the commercial availability of labelled SLs [37]. The synthetic SL analog GR24 (Fig. 1) is structurally the most similar compound to authentic SLs and therefore used by many as an internal standard in SL quantification [20, 38, 39]. Nevertheless, GR24 is more stable than natural SLs in aqueous solutions, which may influence the reliability of the obtained data [16].

Here we report an improved strategy for the efficient extraction and pre-concentration of seven canonical SL representatives, requiring only small initial amounts of root tissue and exudate samples. We have developed a rapid UHPLC separation of these canonical SLs including non-canonical carlactone and carlactonoic acid, followed by highly sensitive MS/MS analysis with detection limits ranging between 125 amol to 2.5 fmol. Validated method facilitates sample preparation, improves analyte stability and allows reliable SL profiling in different plant species and sample backgrounds.

## Results and discussion

### Strigolactone stability in solvents

One of the first and most critical steps in quantitative analysis of compounds with limited stability is the selection of appropriate solvents. With respect to their chemical nature, all SLs are in general adequately soluble in water, aqueous organic solvent mixtures as well as in polar aprotic solvents, like ethyl acetate or DMSO [16, 21, 40]. Employment of water-miscible organic solvents meets compatibility conditions with reversed phase-based (RP) SPE purification prior to final UHPLC–MS/MS analysis. SLs are usually isolated from different sample matrices including a wide range of plant tissue extracts or large volumes of aqueous root exudates [10, 20]. The possibility to change the percentage of organic modifier in the solvent can help to increase the extraction efficiency and eliminate the signal of matrix-associated interfering compounds during LC–MS analysis [32, 33]. Based on these criteria, methanol, acetonitrile and acetone with unlimited water miscibility are the primary solvents of choice. However, the decreased stability of SLs and SL analogues in the presence of nucleophiles has been discussed [15, 17, 19, 37, 41, 42]. Nucleophilic agents like water and methanol can cause a dramatic decrease in SL bioactivity [41]. Therefore, we decided to find the most suitable conditions for SL extraction by testing their chemical stability in water and different aqueous mixtures (5%, 10%, 80%, 100% organic/water, v/v) of three selected organic solvents. The percentage of the organic component in the solvent was chosen to provide efficient solubility of the analytes, SLs, as well as to meet compatibility with loading and elution conditions of RP-based SPE sorbents used afterwards. Triplicates of each solution were spiked with authentic SL standards and then incubated for 3 and 12 h at 0 °C to simulate a fast and a slow extraction procedure, respectively. For individual compounds, the results are represented in four categories as a recovery range 0–10%; 10–50%; 50–90% and 90–100% (Additional file 1: Table S1). A relatively high stability for the tested SLs, with recoveries > 95%, was observed in pure water and aqueous mixtures of all three organic solvents at 5%, 10% and 80% v/v. Overall recovery after 3 h ranged from 96 to 100% and the amount of analyte did not decrease significantly over 12 h (Additional file 1: Table S1). Comparable recoveries were observed for acetone with poor nucleophilicity. However, with prolonged exposure the yield of SLs in pure acetonitrile decreased to 65%. The use of 100% methanol resulted in a 70-80% loss already in 3 h and orobanchol and solanacol were degraded for over 92% within 12 h. The effect of water, 5% methanol/water and 100% methanol on compound recovery after 3 h can also be deduced from the comparison of peak intensities in representative chromatograms (Additional file 1: Figure S1). These SL stability issues in methanol were already previously discussed [16, 19, 37, 40]. Surprisingly, a recently developed UHPLC–MS/MS based method for direct determination of tomato SLs in minute amount of root tissue routinely includes 100% methanol as solubilizing solvent for standard stocks and samples [39]. Our results of SL stability in pure extraction solvents at 0 °C do not support the claims that there is a difference in the life time of GR24 and 5-DS [16, 19], but see below. The ratio of their overall recovery across all solvents and solvent concentrations was 1.08 ± 0.2.

### Strigolactone stability in a sample matrix

**Fig. 2:**
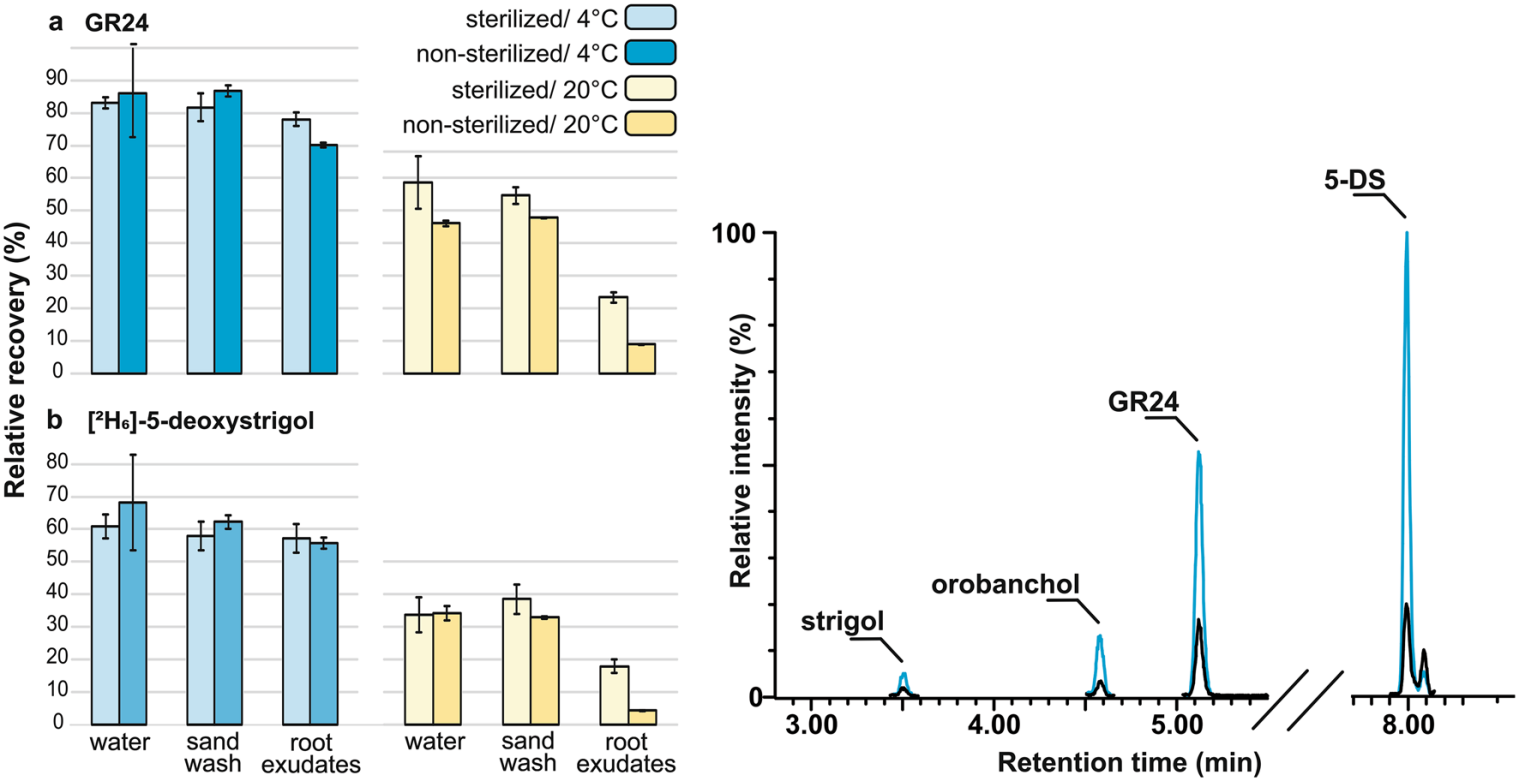
SL stability in sample matrices. The relative recovery of GR24 (a) and [^2^H_6_]-5-DS (b), measured after 8 h in contact with sterilized/non-sterilized water, sand wash and sorghum root exudate at 4 °C (blue bars) and 20 °C (yellow bars). Samples were analyzed in three replicates and error bars represent the standard deviation of the mean (± SD). The UHPLC–MS/MS chromatogram shows the comparison of peak areas assigned to endogenous compounds in non-sterilized and sterilized sorghum root exudates, kept at 4 °C (blue line) and 20 °C (black line), respectively

Another important stability-related issue of quantitative analysis includes the stability of the analyte concentration in sample matrices over time. Direct SL extraction from root tissue by ethyl acetate or freezing of harvested material avoids possible chemical and enzymatic degradation before the sample is processed [20, 22]. Nevertheless, time consuming collection of root exudates at ambient room temperature may possibly cause analyte loss. In order to find out what is critical in the collecting conditions, the effect of temperature on SL recovery in sorghum root exudate samples was analyzed. Standards of GR24 and stable isotope labelled [^2^H_6_]-5-DS were used to spike sorghum root exudates including control wash of the sand substrate without plant, and pure solvent (deionized water). These spiked samples were subsequently stored in a cold room at (4 °C) and at room temperature (20 °C) for 8 h, then further processed and analyzed by UHPLC–MS/MS. Recoveries were calculated against calibrators spiked into dry biological/blank matrix in order to compensate the ion suppression/enhancement effect of matrix background. The storage temperature had a strong impact on the SL lifetime (Fig. 2). In contrast to stability results of SL standards kept at 0 °C in pure solvents, the recovery of GR24 at 4 °C was on average 80.9% (Fig. 2a), about 1.3-fold higher than for [^2^H_6_]-5-DS (Fig. 2b). For both GR24 and [^2^H_6_]-5-DS, recoveries were substantially lower at 20 °C with on average in water 52.3% recovery for GR24 and only 33.8% for [^2^H_6_]-5-DS. While a negligible difference was observed between recoveries in matrix and matrix-free samples at 4 °C, there was a strong negative effect of the root exudate matrix on samples maintained at 20 °C. The nearly 96% loss of [^2^H_6_]-5-DS in sorghum root exudate was slightly reduced by sample sterilization, which also improved the recovery of GR24. However, at 4 °C this did not have a significant effect on the recovery of GR24 nor [^2^H_6_]-5-DS. The endogenous levels of strigol, orobanchol and 5-DS were determined within the dynamic range in sorghum root exudates kept at 4 °C, as well as in filter sterilized samples kept at 20 °C. The peak area of these compounds was reduced over 2.4-fold in the samples maintained at room temperature (Fig. 2, the UHPLC-MS/MS chromatogram). The loss of 5-DS and its labelled counterpart was the same, at about 70%. No decomposition of deuterium labelled standard [^2^H_6_]-5-DS due to back exchange was detected in mass spectra of any of the samples. Although the overall pH of root exudates was slightly higher (7.4) than in control samples (6.5), the similar recovery results obtained at 4 °C exclude a large role for this pH difference in the stability of the tested SLs, although there is a significant effect at 20 °C. Considering the poor SL stability in the root exudate matrix at 20 °C, fast sample collection and processing at a low temperature is recommended.

### Optimization of UHPLC–MS/MS conditions

**Fig. 3:**
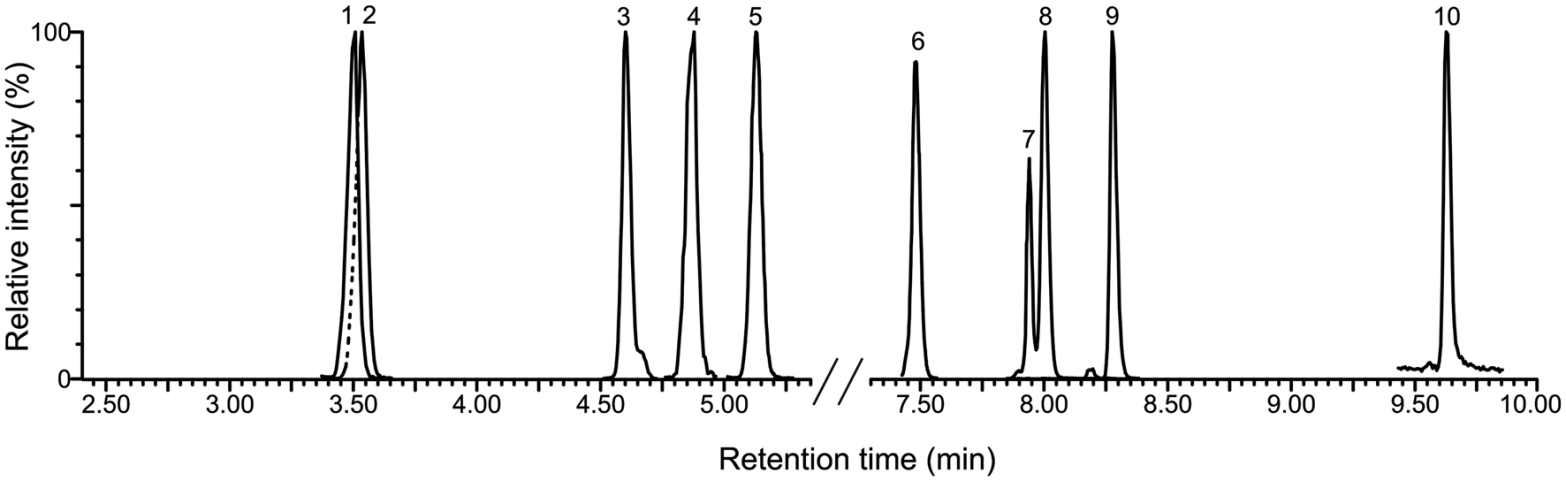
Chromatographic separation of SLs and biosynthetic precursors analyzed by UHPLC–MS/MS. The figure shows normalized MRM chromatograms of strigol (1), solanacol (2), orobanchol (3), sorgomol (4), GR24 (5), sorgolactone (6), 4-deoxyorobanchol (4-DO, 7), 5-deoxystrigol (5-DS, 8), carlactonoic acid (9) and carlactone (10), represented by 0.5 pmol of each analyte injected onto Acquity UPLC^®^ BEH C18 2.1 × 100 mm, 1.7 µm column and separated by gradient elution using 15 mM formic acid in both water and acetonitrile component of mobile phase

The establishment of efficient chromatographic separation can significantly increase final method sensitivity. The UHPLC technology, using columns packed with polymer-based 2-sub-microne particles, greatly improves chromatography resolution and was successfully applied in the analysis of multiple phytohormone classes [33]. While reversed-phase columns containing 1.7 µm particles with low level surface charge (Acquity UPLC^®^ CSH—charged surface hybrid) provide better peak shape and resolution for acidic and basic phytohormones [32, 33], the sufficient separation of SLs, lacking ionizable moieties, can be achieved by uncharged C18-functionalized polymer-based sorbent [13, 20, 30, 39]. For this reason, the Acquity UPLC^®^ BEH C18 was employed in our study. In order to find optimal conditions for UHPLC separation of as many SLs as possible in a single run, the solution of nine authentic SLs including three internal standards was injected on a reversed-phase Acquity UPLC^®^ BEH C18 column (2.1 × 100 mm, 1.7 μm particle size, Waters, Milford, MA, USA). In general, changes in separation efficiency and response (ion intensity) are a function of the mobile phase composition. In terms of SL stability acetonitrile is preferred as an organic modifier. Additionally, we compared three different molar concentrations of formic acid (7 mM, 15 mM, and 25 mM) as the eluent additive in mobile phase. Although the retention of neutral SL compounds showed only little dependence on tested additive concentrations, chromatographic resolution between 5-DS and 4-DO was slightly reduced with 7 mM formic acid in the mobile phase. Nevertheless, the lowest concentration of formic acid improved the sensitivity 1.7-fold compared to the highest (Additional file 1: Figure S2). The composition of the binary mixture was finally optimized at 15 mM formic acid in both water (A) and acetonitrile (B) in order to achieve baseline separation of 5-DS from its 2′-stereoisomer while maintaining the highest possible signal. The gradient elution was programmed to achieve maximal resolution and ionization efficiency of analytes in both positive and negative ESI mode. All compounds were separated within 10 min (Fig. 3, Table 1) and peak retention time reproducibility ranged between 0.01 and 0.02 min. Chromatographic analysis was divided in eight serial scan windows to increase the number of data points across peaks and improve the signal of the targeted ions: 2.8–4; 4–4.8; 4.6–5.1; 4.8–5.5; 7–7.7; 7.6–8.5; 8.1–8.7; 9.3–10 min. Although under our conditions the retention time of solanacol is only about 0.03 min later than that of strigol, their signals acquired under different MRM transitions do not overlap if both are present in the mixture.

**Table 1:** Method parameters

Authentic compound	Retention time^a^ (min)	Scan mode	Precursor ion	Product ion I	Product ion II	Cone voltage (V)	Collision energy (eV)	LOD^b^ (fmol)	Linear range (pmol)	*R*^2^
Strigol	3.50 ± 0.02	+	347	96.9	233	18	15	2.5	0.0125–2.5	0.995
Solanacol	3.54 ± 0.01	+	343	96.9	183	15	20	0.25	0.00125–2.5	0.997
Orobanchol	4.59 ± 0.01	+	347	97	205	15	20	1.25	0.0025–2.5	0.998
Sorgomol	4.85 ± 0.02	+	347	233	133	15	25	2.5	0.0125–2.5	0.992
Sorgolactone	7.48 ± 0.01	+	317.1	96.9	133	20	23	0.125	0.00025–2.5	0.998
4-DO	7.94 ± 0.01	+	331.1	96.9	216	15	18	0.125	0.00025–2.5	0.999
5-DS	8.00 ± 0.01	+	331.1	96.9	216	15	18	0.125	0.00025–2.5	0.999
Carlactonoic acid	8.28 ± 0.01	–	331	113	242	20	12	2.5	0.005–2.5	0.997
Carlactone	9.63 ± 0.02	+	303	96.9	285	15	18	2.5	0.0125–2.5	0.992

Internal standard								Quantified analyte
GR24	5.13 ± 0.01	+	299	96.9	185	20	20	Strigol, solanacol, orobanchol, sorgomol
[^2^H_6_]-2′-*epi*-5-DS	7.88 ± 0.01	+	337	96.9	221	15	18	4-DO
[^2^H_6_]-5-DS	7.94 ± 0.01	+	337	96.9	221	15	18	Sorgolactone, 5-DS

^a^Values are mean ± SD (n = 5); ^b^ The signal-to-noise ratio was set to 3:1

MRM transitions of authentic SLs and corresponding internal standards, including optimized instrument settings and UHPLC–MS/MS method parameters (retention time, limit of detection—LOD, linear range and coefficient of determination—*r*^*2*^)

Most SLs, except carlactonoic acid, lack an ionizable moiety, and were analyzed only in positive ESI mode. Carlactonoic acid was analyzed in negative mode and provided a strong signal as a negative ion [M-H]^−^. All other SLs including the biosynthetic precursor carlactone were weakly ionized, showing the most abundant [M+Na]^+^ ion with fluctuating intensity. Therefore the presence of formic acid in the mobile phase was essential to support formation of protonated parent molecules [M+H]^+^. According to previously published data, the protonated molecular ions [M+H]^+^ and the sodium adducts [M+Na]^+^ are commonly extracted first by the quadrupole and typical transitions to corresponding product ions missing the D-ring are then measured [8, 20, 21, 27]. All biologically active SL-like compounds are characterized by the presence of the butenolide D-ring that gives rise to an identical fragment at *m/z* 97 in positive mode ion-mass spectra. The fragment *m/z* 97 was also observed as predominant product ion after collision induced dissociation of carlactone and most of the other analyzed SL metabolites, in carlactonoic acid D-ring fragment is represented by *m/z* 113 (Table 1). Transitions of precursor-product ion (MRM) with the highest intensity were used as diagnostic. Individual quantifying and secondary qualifying transitions are summarized in Table 1. Mass spectrometer parameters like capillary/cone voltage, collision energy and the number of scans across the peak (dwell time) were optimized for each selected MRM transition to achieve the maximum overall sensitivity.

### Extraction efficiency and optimization of strigolactone purification

Considering the limited stability of SLs as well as their occurrence at very low concentrations in both root tissue and rhizosphere (10^−12^–10^−15^ molar per gram of fresh root weight), the development of a rapid and efficient enrichment strategy is a prerequisite [27, 43]. Current methods that process sample of ≥ 0.5 l exudate mostly combine pre-concentration on low-specific RP SPE sorbents with additional analyte purification using normal phase (NP) SPE. Root tissue samples (≥ 200 mg of fresh weight) are usually first extracted with water immiscible ethyl acetate before Silica-NP-SPE clean-up [22, 44–46]. Compared to silica-based SPE materials, polymeric RP SPE is characterized by higher stability and sample capacity due to the larger surface area, making them highly suitable for extraction of plant hormones from minute sample amounts [32, 33, 47]. For SL isolation from aqueous extracts, we decided to evaluate four different commercially available RP SPE materials. The extraction capacity of two silica-based (Strata^®^ C18-U and Strata^®^ CN, Phenomenex) and two polymer-based (Strata^®^ X, Phenomenex; and Oasis^®^ HLB, Waters) solid phases was tested on a background of pure solvent as well as sorghum root exudate matrix. In order to achieve sufficient selectivity throughout the SL spectrum, the retention of solanacol, GR24 and [^2^H_6_]-5-DS, together covering a broad range of SL polarity, was tested, also because they are not naturally produced by sorghum. Samples of 20 ml were prepared in 10% aqueous acetonitrile to assure all analytes quantitatively dissolved. Liquid samples were spiked with the mixture of SL standards and loaded onto pre-conditioned columns. SLs were eluted with aq. 80% acetone. Eluates were dried and reconstituted in initial mobile phase for UHPLC–MS/MS analysis. The sorbent of Oasis^®^ HLB (Waters) and Strata^®^ X (Phenomenex), having a dual retention mechanism based on both hydrophilic and lipophilic interactions, showed less than 10% loss in total SL recovery when loaded in absence of sample matrix (Additional file 1: Table S2). While equally good analyte recoveries (≥ 86%) were achieved after Oasis^®^ HLB purification of root exudates, polymer Strata^®^ X provided, in total, 1.5-fold lower recovery than HLB and irreproducible results (RSD% ≥ 16). Compared to the polymer HLB material, the silica-based C18 stationary phase showed a slightly lower recovery for the more polar solanacol (around 75%). Although bigger volumes of aqueous flow-through and washing fractions were not analyzed for SL presence, the examined RP endcapped -CN sorbent for interactions with moderately polar compounds appeared ineffective to retain the more hydrophobic [^2^H_6_]-5-DS (75 ± 1.0%) already in the background of pure solvent. Moreover, the presence of root exudate matrix decreased the recovery of all three spiked internal standards, varying according to their polarity with the highest losses for [^2^H_6_]-5-DS (22 ± 2.0%). Based on the obtained data we selected the Oasis^®^ HLB columns packed with 150 mg of macroporous copolymer [poly-(divinylbenzene-co–*N*-vinylpyrrolidone)] for further development of the extraction protocol.

The extraction of SLs from plant root tissue was optimized using acetone-based solvents. Although acetone provides a good solubility and stabilizes a broad range of SLs, co-extracted interfering plant compounds can complicate further SL SPE pre-concentration as well as their analysis by LC–MS/MS due to high chemical background. In order to efficiently suppress the background signals of the matrix an increase in the percentage of water in the extraction solvent can be used [32, 47]. The extraction efficiency of 80% and 60% acetone aqueous mixtures (v/v) was tested on sorghum root, cv. Shanqui red grown under low-phosphate conditions. Two hundred mg of root tissue ground in liquid nitrogen, was further homogenized using a tissue-lyzer in the presence of extraction solvent and internal standard, and was sonicated to adequately release the SLs from the tissue. The acetone fraction of the extraction solvent was evaporated in *vacuo* and the residual water in the sample was made up to 4 ml to achieve 10% acetonitrile/water (v/v). Samples were further purified using polymer-based Oasis^®^ HLB columns as described above and analyzed by UHPLC–MS/MS. In both 60% and 80% acetone extracts of fresh sorghum root tissue equal endogenous levels of 5-DS were detected (1.24 ± 2% and 1.19 ± 12% pmol/g, respectively; values are means ± RSD%, n = 3). Moreover, recovery values of the labeled standard [^2^H_6_]-5-DS were approximately 73% and showed no significant difference in matrix effect using both extraction solvents (data not shown). The low detection and quantification limits of the optimized UHPLC–MS/MS method allowed us to reduce the initial weight of sample material to 150 mg. The decrease in sample size significantly improved the recovery of the internal standard to 89.6 ± 1% while the quantified levels of 5-DS recalculated per gram of fresh weight remained similar (Table 3).

In order to reduce the initial sample volume of root exudates, we tested whether addition of an organic modifier to the water used to flush the pots can improve SL extraction efficiency from sand substrate. The SL presence and possible differences in yield were confirmed in root exudates of a relative high SL producer, rice variety Shiokari. In each individual pot filled with silver sand, four rice seedlings were grown under low phosphate conditions. Biological quadruplicates of rice root exudates were separately washed from the rhizosphere using deionized water and an aqueous 5% solution of acetonitrile (v/v). Regardless of the volume of the extraction solvent added to the three-liter pots, the first 200 ml of flow-through fraction was collected. Only 20 ml of the total sample volume was further purified, in order to avoid exceeding the SPE sorbent capacity, and analyzed for SL content by the optimized UHPLC–MS/MS method. The 4-DO, endogenously produced in the rice variety Shiokari [2, 31], was detected in both types of root exudate extracts. Additional file 1: Figure S3-b shows the comparison of compound levels in 200 ml of collected solvent. In contrast to water, around 2.7-fold higher 4-DO yield was achieved due to better elution strength of 5% acetonitrile/water (v/v, Additional file 1: Figure S3). An additional 200 ml rhizosphere wash with 5% acetonitrile contained 4-DO at the detection limit (data not shown). Using the organic modifier in the extraction solvent was beneficial for an efficient analyte enrichment from the rhizosphere of sand-grown plants. It also decreased the initial sample volume (20 ml) by more than 25-fold compared to previously published procedures [48–50]. Moreover, single-step purification of low volumes of root exudates significantly reduces the sample preparation time to < 30 min or < 3 h including the final evaporation step. This facilitates the processing of larger sets of samples, compared to conventional methods where time-consuming concentration of exudates (> 500 ml) and subsequent purification is performed using two-step SPE, mostly requiring 2 days [45, 50, 53, 54]. Additionally, low volumes can be more easily kept at a low temperature, thus improving the stability of SL analytes. For practical reasons an alternative to the toxic acetonitrile, such as ethanol at the same percentage, could be used as extraction solvent for root exudates. Although pure primary alcohol has a negative effect on SL stability, SL recovery in ≤ 80% aqueous solutions of methanol ranged between 90 and 100% for 12 h (Additional file 1: Table S1).

**Fig. 4:**
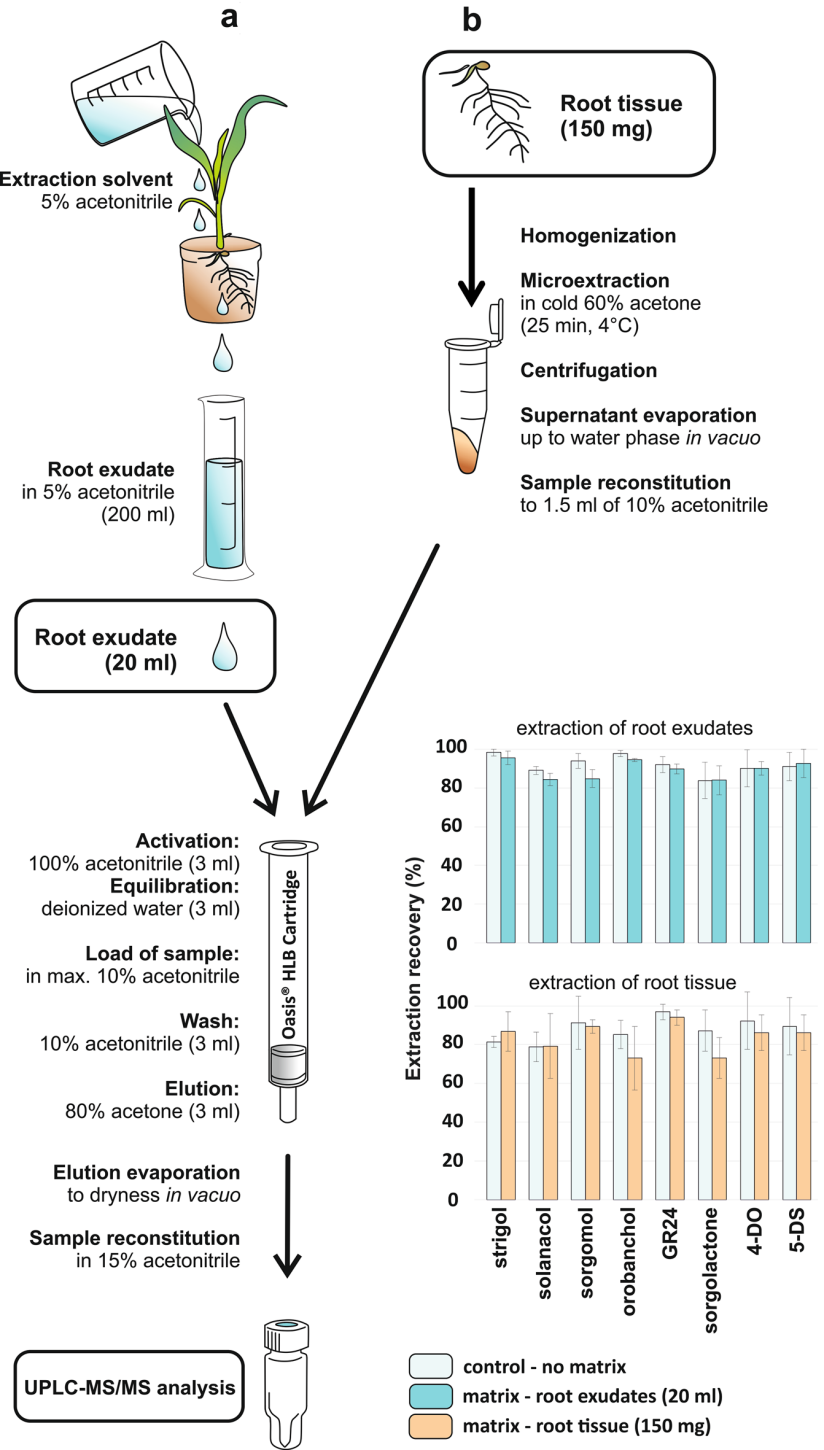
Optimized protocol for strigolactone extraction and purification from root exudate (**a**) and root tissue (**b**). The bar chart represents the extraction recovery of individual compounds spiked in samples of root exudates (20 ml) and root tissue (150 mg of fresh weight) including matrix-free extraction solvent (control). Samples were analyzed in three replicates. Error bars represent the standard deviation of the mean (± SD)

The extraction recovery of eight selected SLs was evaluated in the background of sorghum root exudates and root tissue. Both SL precursors – carlactone and carlactonoic acid were excluded from this experiment as well as further extraction validation due to the lack of required amount of authentic standard. For exudates, 20 ml of collected volume was spiked with SL standards at 5 pmol. The same amount was added to ground root tissue prior to extraction (Fig. 4). Relatively good recoveries ranging from 83.8 – 98.2% were obtained for root exudates and values did not significantly differ from compounds purified in a background of control solvent without sample matrix (Fig. 4). The protocol for extraction from root tissue includes some additional step of sample evaporation and reconstitution prior to SPE which may be the reason for the increase in the standard error. Nevertheless, the average SL loss was only 13% in matrix-free samples and 16.1% in root tissue extracts.

### Method validation

Highly precise and accurate MS-based quantitation of SLs is strongly dependent on molecule stability and sample matrix. Behavior of endogenous compounds during sample preparation, ionization and detection can be normalized by addition of stable isotope labeled analogues in known concentration. However, commercial availability of suitable SL standards is limited by their complicated synthesis [37]. Therefore, we studied the possibility to use the SL structural analogue GR24, which is not naturally present in any plant species, as internal standard. Hereto, first the linearity for each SL was assessed by triplicate injection of the set of standards with 12 variable levels of analyte and one fixed concentration of internal standard, GR24. The resulting data were plotted after logarithmic transformation. To allow for correction for stability differences between synthetic GR24 and authentic SLs in the calibration mixture, the peak area ratio of analyte to internal standard in eight calibration points was monitored in duplicate every 7 days over 3 weeks. A new calibration mixture was freshly prepared and injected for each time point to normalize instrument sensitivity response. The UHPLC–MS/MS method showed great linearity for SL compounds eluted in the first half of the gradient when using GR24 as internal standard. As summarized in Table 1, the correlation coefficient (*r*^*2*^) values of strigol, solanacol, orobanchol and sorgomol varied between 0.992 and 0.998 within a concentration range of at least three orders of magnitude. We found lower *r*^*2*^ values (0.97–0.98) for the less polar sorgolactone, 4-DO and 5-DS with GR24 likely as a result of differences in stability and chromatography (data not shown). Therefore, stable isotope labeled standards [^2^H_6_]-2′-*epi*-5-DS and [^2^H_6_]-5-DS were used to correct recoveries of these SL metabolites (Table 1). Unfortunately, none of the internal standards were suitable for quantitation of carlactone and carlactonoic acid, likely as a result of dissimilar chemical structure, stability or ion detection in opposite mode. Their analytical linearity was therefore measured without the response of an internal standard (Table 1). To evaluate sensitivity parameters, the instrumental limits of detection (LOD) were estimated from individual chromatograms of standard solutions as signal to noise (S/N) ratio of 3:1. Detectable concentration levels were lower than 2.5 fmol (Table 1) and maximal S/N ratio was obtained for sorgolactone, 4-DO and 5-DS with a LOD close to 125 amol. A similar LOD for orobanchol in pure solution background was also achieved by Boutet-Mercey et al. Our detectable orobanchol amount of 1.25 fmol is only about two-fold lower than the reported concentration [20]. Compared to previously published work, the combination of the efficient analyte isolation with the excellent method sensitivity allows SL analysis in 3.3 to 25 times lower initial amounts of root tissue and root exudate volumes, respectively [20, 22, 51].

The optimized UHPLC–MS/MS-based method for SL profiling was further validated in terms of precision and accuracy [52]. Representative sample matrices (150 mg of fresh weight sorghum root tissue; 20 ml of sorghum root exudates) and corresponding matrix-free extraction solvents were spiked with authentic SL metabolites in two concentrations (1 and 10 pmol) during the extraction procedure. Final quantitation of endogenous compounds in samples was estimated by standard isotope dilution analysis using peak area ratio of authentic to internal standard. The intra-day method precision, expressed as a percentage of relative standard deviation (RSD%), was assessed from quadruplicate measurements of each concentration. The precision at both concentrations ranged between 1.0–5.3% in root exudates and 0.4–12.3% in root tissue extracts (Table 2). The analytical accuracy determined as a percentage bias (% bias) at 1 pmol ranged between − 6.8–5.8% in root exudates and 0.2–1.3% in root tissue extracts. Slightly lower accuracy was observed in samples of root exudates, ranging between − 12.2 and − 2.9%, and root tissue extracts, − 12.8–2.0%, after standard spiking with 10 pmol. A similar SL yield in matrix containing samples was achieved in control samples of spiked extraction solvent (Additional file 1: Table S3). The overall precision/accuracy means were 2/− 2.01 and 2.7/− 3.58 for the root exudate and tissue extraction procedure, respectively. Validated parameters within an acceptable range showed that our optimized extraction and purification strategy, followed by UHPLC–MS/MS-based analysis is suitable for simultaneous profiling of seven SL metabolites in two different sample matrices.

**Table 2:** Method validation

Compound	Determined spiked content (pmol)^a^	Method precision (%RSD)^a^	Method accuracy (%bias)^a^	Determined spiked content (pmol)^b^	Method precision (%RSD)^b^	Method accuracy (%bias)^b^
Root exudates (20 ml)						
Strigol	1.03 ± 0.03	3.0	3.45	9.71 ± 0.20	2.0	− 2.94
Solanacol	0.95 ± 0.03	2.7	− 4.90	8.78 ± 0.12	1.3	− 12.21
Orobanchol	0.93 ± 0.05	4.9	− 6.89	9.42 ± 0.10	1.0	− 5.78
Sorgomol	1.03 ± 0.05	5.3	2.83	9.28 ± 0.14	1.5	− 7.23
Sorgolactone	1.03 ± 0.05	5.3	2.83	9.28 ± 0.14	1.5	− 7.23
4-DO	1.06 ± 0.04	3.7	5.89	9.57 ± 0.24	2.5	− 4.32
5-DS	1.03 ± 0.04	3.6	2.79	9.39 ± 0.15	1.6	− 6.08
Root tissue (150 mg)						
Strigol	1.07 ± 0.05	4.4	0.67	9.81 ± 0.12	1.2	− 1.91
Solanacol	1.05 ± 0.04	3.4	0.47	8.72 ± 0.09	1.0	− 12.83
Orobanchol	1.09 ± 0.04	3.8	0.92	9.72 ± 0.25	2.6	− 2.80
Sorgomol	1.13 ± 0.14	12.3	1.33	10.12 ± 0.04	0.4	1.18
Sorgolactone	1.07 ± 0.04	3.3	0.66	9.70 ± 0.15	1.6	− 2.95
4-DO	1.08 ± 0.01	1.2	0.81	9.78 ± 0.09	0.9	− 2.15
5-DS	1.05 ± 0.01	0.9	0.46	10.20 ± 0.13	1.2	2.05

The precision and accuracy evaluated by spiking a fixed amount of sorghum root exudate (20 ml) and root tissue (150 mg of fresh weight) with analytes at two different concentrations (^a^ 1 pmol, and ^b^ 10 pmol) prior to individual extraction procedures. Values are mean ± SD (n = 4)

### Strigolactone quantitation in root tissue and exudates of plants

In order to further test our newly established purification and analysis protocol, we performed SL analysis in real samples of four different agriculturally important plant species and compared the obtained results with published data. Growing plants under phosphate deficiency has been reported many times as a stimulating factor for both SL biosynthesis and exudation [9, 43, 51] and we therefore used that condition to achieve measurable concentration levels of SLs.

Three different varieties of Asian rice (*Oryza sativa* L.) were analyzed and compared for SL content. The pathway from the SL precursor carlactone to the canonical SLs, 4-DO and from there to orobanchol in rice has been reported [31, 35, 53]. Our results also confirmed the major presence of 4-DO and orobanchol, with the highest abundance per gram of root weight especially in the cultivar Shiokari that is highly susceptible to infection by the parasitic weed, *Striga hermonthica*, of which germination is induced by SLs (Table 3). In contrast, the high-quality and disease resistant *indica* variety IR 64 exuded only femtomolar level of orobanchol-type SLs. The signal of 4-DO in exudates of drought tolerant rice cv. Apo was under the limit of quantification. No SLs were detected in the roots of both *indica* subspecies, but the roots of Shiokari contained a clearly detectable amount of 4-DO. Similar differences in SL concentration levels between *Japonica* rice Azucena and *Indica* cultivar Bala were reported [53].

**Table 3:** Strigolactone content analyzed by UHPLC-ESI–MS/MS

	Cultivar	Strigol		Solanacol		Orobanchol		Sorgomol		Sorgolactone		4-DO		5-DS	
		E	R	E	R	E	R	E	R	E	R	E	R	E	R
*Oryza sativa L.*	Shiokari	–	–	–	–	0.20 (± 11)	–	–	–	–	–	2.03 (± 5)	7.79 (± 9)	–	–
	IR 64-21	–	–	–	–	*LOD*	–	–	–	–	–	0.59 (± 12)	–	–	–
	APO	–	–	–	–	–	–	–	–	–	–	*LOD*	–	–	–
*Sorghum bicolor*	Shanqui red	0.44 (± 16)	–	–	–	0.02 (± 16)	–	–	–	0.02 (± 10)	–	–	–	1.62 (± 22)	1.03 (± 8)
	SRN 39	0.04 (± 19)	–	–	–	9.65 (± 18)	4.42 (± 10)	0.44 (± 13)	*LOD*	0.09 (± 15)	–	–	–	0.004 (± 36)	–
*Pisum sativum* L.	Sprinter	–	–	–	–	0.27 (± 11)	1.31 (± 17)	–	–	–	–	–	–	–	–
	RoR12	–	–	–	–	0.11 (± 18)	1.37 (± 13)	–	–	–	–	–	–	–	–
*Solanum Lycopersicum L.*	MoneyMaker	–	–	1.26 (± 17)	2.26 (± 5)	0.83 (± 18)	1.08 (± 8)	–	–	–	–	–	–	–	–

Concentrations levels of endogenous strigolactones per gram of fresh root weight, analyzed in root exudates (E) and root tissue (R) of different plant species and varieties

*LOD* level on limit of detection, *–* not detected

^a^Values are means (± RSD, %, n = 4)

The results obtained from SL profiling in sorghum roots and exudates confirmed the differences between the 5-DS producing genotype Shanqui Red and the low *S. hermonthica* germination stimulant, mostly orobanchol producing SRN39 [50, 54]. In Shanqui red, strigol-type SLs represented 99% of the total amount of SLs in the root exudate, with 5-DS being the most abundant and the only one detectable in root tissue (Table 3). Orobanchol was the major SL in both root extracts and exudates of SRN 39, and the amount analyzed in collected exudates was approx. 171 pmol per average root mass in pot. Other exuded compounds such as strigol, 5-DS, sorgomol and sorgolactone accounted for less than 6% of the total SL content in SRN 39 samples.

Orobanchol-type SLs are also commonly present in root exudates of pea (*Pisum sativum* L.) [20, 39, 45]. While the endogenous levels of orobanchol in both root tissues were comparable, the exudates of Sprinter contained a twofold higher amount of orobanchol compared to the low-strigolactone line RoR12 (Table 3). An almost ten-fold difference in orobanchol content between both cultivars was observed by Pavan et al. after 8 days of phosphate starvation by analyzing 5 L of root exudates (~ 10 pmol/sample of cv. Sprinter).

In tomato MoneyMaker we analyzed the previously reported orobanchol and solanacol [6, 55, 56] in both root exudate and root extracts (Table 3). The concentration level for these two SLs in root exudate was comparable. In root extracts the absolute amount of endogenous solanacol was more than two-fold higher than for orobanchol, which is slightly different from Zhang et al. [56] who reported a higher orobanchol than solanacol level in their tomato genotype. Nevertheless, the plant age, growing conditions as well as duration of phosphate starvation might play a role in final SL profile.

Biosynthetic precursors of SLs, carlactone and carlactonoic acid, were not detected in any of analyzed root tissue. Due to low concentration levels and instability, these compounds are usually isolated from expression platforms as products of yield-boosting enzymes of SL biosynthesis [56–58]. Ten times higher amount of root tissue was necessary to successfully determine the endogenous SL precursors in roots of rice and *Arabidopsis* [31, 58].

## Conclusions

Strigolactones are a relatively new, hot topic in the field of plant hormone research. Their analysis is compromised by limited stability and low concentrations in sample matrices. In this report, we present a highly sensitive and validated UHPLC–MS/MS method for simultaneous profiling of seven naturally occurring canonical strigolactones in some of the most frequently studied plant materials—root tissue and exudates. In order to minimize the strong matrix effect, the sample size of root exudate extracts and roots was reduced to 20 ml and 150 mg, respectively. A combination of rapid extraction with water miscible aprotic solvents and efficient single-step purification using macroporous polymer-based sorbents with both hydrophilic and lipophilic retention characteristics resulted in a fast and simple sample preparation procedure. This notably improved stability and extraction recovery of selected SLs (73.1–95.6%). For targeted UHPLC–MS/MS analysis a method was developed in which a range of different SLs including their biosynthetic precursors can be separated within a 10 min retention time frame using an analytical column packed with sub-2-microne particles. The detection limits for these SLs range from 0.125 to 2.5 fmol with a linearity of three to five orders of magnitude. Seven SLs were successfully detected and quantified in a series of very different, real plant-derived samples showing the versatility of the developed method.

## Methods

### Standards and reagents

The SL standards (±)-strigol, *ent*-2′-*epi*-5-deoxystrigol (4-DO), (±)-5-deoxystrigol (5-DS), (±)–orobanchol, (−)-sorgomol, (±)-solanacol and (+)-sorgolactone were kindly provided by prof. Binne Zwanenburg (Radboud University Nijmegen, The Netherlands), prof. Koichi Yoneyama (Utsunomiya University, Japan) and prof. Yukihiro Sugimoto (Kobe University, Japan). The stable isotope labelled internal standards [^2^H_6_]-2′-*epi*-5-deoxystrigol and [^2^H_6_]-5-deoxystrigol were generous gifts from prof. Tadao Asami (The University of Tokyo, Japan). Natural carlactone was prepared and kindly provided by Dr. Adrian Scaffidi (The University of Western Australia, Australia). The standards of (±)-GR24 and carlactonoic acid were a gift from prof. Alain de Mesmaeker (Syngenta, Switzerland). Deionized water for aqueous extraction solutions was obtained from a Simplicity^®^ UV Water Purification System (Millipore, Merck, Bedford, MA, USA). Other solvents such as methanol, acetone, eluent additive formic acid, acetonitrile and water for UHPLC–MS/MS analysis were of HPLC-grade or higher purity purchased from Merck KGaA (Darmstadt, Germany). Solid chemicals of analytical grade were from Sigma-Aldrich (Steinheim, Germany).

### SL stability experiments

The mixture from standard solutions of individual SLs was transferred to new vials in aliquots, containing known amounts of analytes (5 pmol per sample). Samples in triplicates were evaporated to dryness *in vacuo* (SPD121P SpeedVac, Thermo Fisher Scientific, Waltham, MA, USA) and dissolved in testing solutions using sonication for 3 min under cooling with ice water (Branson 3510 ultrasonic bath, Branson Ultrasonic, Eemnes, The Netherlands). For testing of SL chemical stability, compounds were incubated with 1 ml of working solutions—5%, 10%, 80%, 100% methanol, acetonitrile, acetone and deionized water—over 3 and 12 h at 0 °C. The stability of SLs in a sample matrix was tested in the background of normal or filter-sterilized sorghum root exudates including control samples of deionized water and water drained through the empty sand substrate on which sorghum was grown. Compounds were in contact with 3 ml of sample over 8 h at 4 and 20 °C, representing different sample collection and processing conditions. Sterilization of water and root exudates was achieved by filtration through a sterile 25 mm nylon syringe membrane filter (Nalgene™, Thermo Fisher Scientific, Waltham, MA, USA) with 0.2 µm pore size. All samples were evaporated to dryness as described above, reconstituted in 100 μl of 15% acetonitrile/water (v/v), filtered using a micro-spin nylon filter (0.45 μm pore size, Thermo Fisher Scientific, Waltham, MA, USA) and analyzed by UHPLC–MS/MS (0.25 pmol per injection). Recoveries were calculated by comparing the results with peak areas of SL standards spiked into dry control as well as matrix samples, in order to compensate ionization yield in ESI due to possible matrix effect.

### Plant material

Plant and root exudate material of *Sorghum bicolor* L. (cvs Shanqui red and SRN39) was used in most of the stability, recovery and validation experiments and generated as follows. Sorghum seeds were sterilized with 2% bleach for 30 min, rinsed with water and kept on moist filter paper in a Petri dish for 48 h/28 °C to induce germination. Germinated seeds were transferred to 1 L pots filled with silver sand. Three sorghum seedlings per pot were grown in a climate chamber under short day conditions at a light intensity of 450 μE m^−2^  s^−1^ (10 h light, 28 °C/14 h dark, 25 °C) and watered each 48 h using half strength modified Hoagland’s solution [54]. After 5 weeks, any remaining nutrients were flushed from the sand using 1 L of deionized water and phosphate deficient half strength Hoagland’s solution (nutrient solution without K_2_HPO_4_ and potassium compensation in the form of KNO_3_) was then used for 1 week to induce phosphate starvation and hence SL production. Compounds accumulated in the sand during this 1 wk were removed by flushing with 1 L of phosphate deficient Hoagland’s solution and 48 h later root exudates, containing newly produced SLs, were collected as detailed below. Subsequently, roots were collected from the sand, weighed, frozen in liquid nitrogen and kept at − 80 °C until further use.

In addition to sorghum, several other plant materials were used in SL profiling experiments. Optimized growing conditions for SL analysis in *Oryza sativa* (cv. Shiokari, IR64, Apo) are described elsewhere [48]. Seeds of *Pisum sativum* L. (cv. Sprinter, RoR12) were obtained from the Department of Soil, Plant and Food Science of the University of Bari (Italy). Seeds were sterilized with 4% sodium hypochlorite containing 0.02% (v/v) Tween 20, washed with deionized water and germinated for 5 d on moistened filter paper at 25 °C in darkness. Seeds were transferred to pots with silver sand as described above, and grown under long day conditions (16 h light, 21 °C/8 h dark, 21 °C; light intensity 450 μE m^−2^ s^−1^). Seedlings were watered each 72 h with Hoagland’s solution [6]. Four wks after sowing, phosphate starvation was applied as described above, and samples of roots and exudates were collected 8 d later. Tomato (*Solanum lycopersicum*, cv. MoneyMaker) plants were grown hydroponically on half strength Hoagland’s solution as optimized previously [6]. Three wk old seedlings were placed individually on 100 ml of Hoagland’s solution without phosphate and after 96 h, tomato root tissue and exudates were collected, and samples of 5 seedlings were pooled into one biological replicate. Nutrient solution was supplied daily in order to keep the volume constant.

### Extraction of root exudates

For method development and SL quantitation analysis, root exudates from sand-grown plants (sorghum, rice, pea) were extracted using a 5% aqueous solution of acetonitrile (v/v). Regardless the exact amount of extraction solvent added to each pot, the first 200 ml of flow-through volume was collected. Twenty ml aliquots of the obtained exudate were transferred to 50 ml Falcon tubes and centrifuged (4.000 rpm/5 min/4 °C) in order to remove solid particles. The nutrient solution of hydroponically-grown tomato seedlings was filtered using Whatman^®^ Cellulose filter paper (Sigma-Aldrich, Steinheim, Germany). In this case, 95 ml of 500 ml total sample volume was collected, to which 5 ml acetonitrile was added. Exudates intended for quantitation were spiked with 5 pmol of internal standards—GR24, [^2^H_6_]-2′-*epi*-5-DS, [^2^H_6_]-5-DS—vortexed and further processed by SPE.

### Extraction of root tissue

Frozen root tissue was homogenized to a fine powder in liquid nitrogen using a mortar and pestle. 150 mg of this powder was transferred to a 2 ml safe-lock Eppendorf micro-extraction tube. Samples were extracted with 1.5 ml of 60% acetone/water (v/v), containing 2-mm yttria stabilized zirconium oxide beads (Next Advance, Inc., Troy, NY, USA) and 5 pmol of internal standards (GR24, [^2^H_6_]-2′-*epi*-5-DS, [^2^H_6_]-5-DS) in order to control for the extraction recovery. Additional homogenization was performed using a bead mill—Tissue-Lyzer II (Qiagen Sciences, Inc., Germantown, MD, USA) at 27 Hz for 3 min. Subsequently, samples were sonicated for 3 min in a Brandson 3510 ultrasonic bath under cooling with ice water and shaken for 30 min in a cold room (4 °C) using a Loopster benchtop laboratory rotator (IKA^®^-Werke GmbH & Co. KG, Staufen, Germany) at 20 rpm. After centrifugation (16.000 rpm/10 min/4 °C), the supernatant was collected and the organic solvent was removed *in vacuo* (Thermo Fisher Scientific SPD121P SpeedVac, Whaltman, MA, USA). The remaining aqueous phase (approx. 0.9 ml) was made up to 1.5 ml by addition of 25% acetonitrile/water (v/v) in order to quantitatively dissolve SL analytes and achieve suitable SPE loading conditions (5–10% acetonitrile/water, v/v).

### Sample purification

The extracts were further pre-concentrated using polymeric reverse phase-based Oasis^®^ HLB columns (150 mg/3 ml, Waters, Milford, MA, USA). The sorbent was activated with 3 ml of 100% acetonitrile and equilibrated with 10% aqueous acetonitrile (v/v). Samples of exudates and root tissue extracts were passed through the activated column. Salts and very polar compounds, not retained by the sorbent, were washed out with additional 3 ml of 10% aqueous acetonitrile (v/v). SL analytes were quantitatively eluted from the column with 3 ml of 80% aqueous acetone and evaporated to dryness *in vacuo*. Dry samples were stored at -20 °C until LC–MS analysis. The same protocol was applied for different types of sorbents to test the extraction capacity for neutral SL analytes. Because the polymeric sorbents—Strata^®^ X (Phenomenex, Torrance, CA, USA) and Oasis^®^ HLB—have a higher surface area and approx. 4-times higher loading capacity, we used a higher sorbent mass for silica-based cartridges Strata^®^ C18-U and Strata^®^ CN (both from Phenomenex, Torrance, CA, USA) of 500 mg/3 ml.

### UHPLC–MS/MS conditions

The Waters Acquity UPLC™ I-Class System (Waters, Milford, MA, USA) equipped with Binary solvent manager and Sample manager was employed as a chromatographic system coupled to a Xevo^®^ TQ-S tandem quadrupole mass spectrometer (Waters MS Technologies, Manchester, UK) with electrospray (ESI) ionization interface. Dry samples were reconstituted in 100 μl of initial mobile phase—15% acetonitrile/water (v/v) and filtered using a micro-spin nylon filter (0.45 μm pore size, Thermo, Waltham, MA, USA). A sample volume of 5 μl was injected onto an Acquity UPLC™ BEH C18 column (2.1 × 100 mm, 1.7 μm particle size, Waters, Milford, MA, USA), maintained at a constant temperature of 45 °C at a flow rate of 0.45 ml min^−1^. The eluents employed were 15 mM formic acid in both water (A) and acetonitrile (B). The elution profile was set as follows: isocratic elution for 0.3 min at 15% of B followed by a consecutive linear gradient increase to 27, 40 and 65% of B in 0.6, 5 and 8 min, respectively. The logarithmic gradient elution to 80% of B was performed until 8.3 min after which the composition was kept constant until 10 min. Then the column was washed with 90% B and equilibrated for initial conditions in 2.5 min. The eluate was introduced in the ESI ion source of the tandem mass analyzer operating at the following conditions: capillary/cone voltage (3 kV/15-20 V); source/desolvation temperature (150/550 °C); cone/desolvation gas flow (150/650 L h^−1^), collision energy (10-23 eV), collision gas flow (0.08 ml min^−1^). Mass data of SLs and their internal standards were acquired in multiple reaction monitoring (MRM) mode (Table 1). The chromatographic run was separated in 8 retention windows (2.8–4; 4–4.8; 4.6–5.1; 4.8–5.5; 7–7.7; 7.6–8.5; 8.1–8.7; 9.3–10 min) and dwell time of each MRM channel was calculated to achieve 16-20 points per peak. The MassLynx™ software, version 4.1 (Waters), was used to control instrument and acquire and process MS data.

## Supplementary information

**Additional file 1: Figure S1.** The comparison of peak intensity between representative chromatograms of authentic SL standards, reconstituted in 20% acetonitrile/water and injected at 0.5 pmol/5 µl at time 0 (a); and standards, incubated for 3 h in pure water (b), 5% methanol/water (v/v, c) and 100% methanol (d). Compounds eluted from the Acquity UPLC^®^ BEH C18 2.1 × 100 mm, 1.7 µm column at the following retention times (min): strigol (3.50 ± 0.01), solanacol (3.53 ± 0.01), orobanchol (4.58), sorgomol (4.86 ± 0.01), GR24 (5.12), sorgolactone (7.47; the standard mixture contains the non-natural isomer of sorgolactone, which eluted at 7.39), 4-deoxyorobanchol (7.93) and 5-deoxystrigol (7.99). **Figure S2.** Comparison of peak intensity and peak-to-peak resolution between authentic standards of 4-deoxyorobanchol and 5-deoxystrigol. The standard mixture in a concentration of 0.5 pmol/5 µl was injected onto an Acquity UPLC^®^ BEH C18 2.1 × 100 mm, 1.7 µm column and separated by gradient elution using 7 mM (a), 15 mM (b) and 25 mM (c) of eluent additive formic acid in the mobile phase. **Figure S3.** Comparison of peak areas of endogenous 4-deoxyorobanchol extracted from rice root exudate with water (blue line) or 5% acetonitrile/water (v/v, black line). Samples were purified by Oasis^®^ HLB (Waters) and analyzed by UHPLC-ESI (+)-MS/MS using the conditions described in Table 1. The bar chart compares the compound concentration calculated in 200 ml of exudate, extracted with the two different extraction solvents. Samples were analyzed in three replicates. Error bars represent the standard deviation of the mean (± SD). **Table S1.** Strigolactone stability in solvents. The stability of individual analytes in water, selected organic solvents and their aqueous solutions at 0 °C during 3 (a) and 12 h (b). The results are represented in four categories of recovery range: - (0-10%), + (10-50%), ++ (50-80%), +++ (80-100%). Values are mean ± SD (n = 3). **Table S2.** Extraction recoveries (%) of selected strigolactones after pre-concentration with different solid-phase materials. The standard mixture containing 5 pmol of solanacol, GR24 and [^2^H_6_]-5-deoxystrigol was loaded onto solid-phase materials in the presence of sorghum root exudate (matrix) and matrix-free extraction solvent (control). The recovery (%) of added compounds was analyzed in the eluent. Values are mean ± SD (n = 3). **Table S3.** Method validation. The analytical precision and accuracy, evaluated by spiking root exudate- and root tissue-free control extraction solvents with analytes at two different concentrations (1 pmol – a, and 10 pmol – b) prior to extraction. Values are mean ± SD (n = 4).

## Data Availability

Datasets analysed and processed for this study are available from the corresponding author on reasonable request.
