# Supplementary material for: An improved strategy to analyse strigolactones in complex sample matrices using UHPLC–MS/MS
Source: Plant Methods. 2020 Sep 17;16:125. doi: 10.1186/s13007-020-00669-3 (PMC7499983; doi:10.1186/s13007-020-00669-3)
Supplement: Supplementary file 1 — Additional file 1: Figure S1. The comparison of peak intensity between representative chromatograms of authentic SL standards, reconstituted in 20% acetonitrile/water and injected at 0.5 pmol/5 µl at time 0 (a); and standards, incubated for 3 h in pure water (b), 5% methanol/water (v/v, c) and 100% methanol (d). Compounds eluted from the Acquity UPLC® BEH C18 2.1 × 100 mm, 1.7 µm column at the following retention times (min): strigol (3.50 ± 0.01), solanacol (3.53 ± 0.01), orobanchol (4.58), sorgomol (4.86 ± 0.01), GR24 (5.12), sorgolactone (7.47; the standard mixture contains the non-natural isomer of sorgolactone, which eluted at 7.39), 4-deoxyorobanchol (7.93) and 5-deoxystrigol (7.99). Figure S2. Comparison of peak intensity and peak-to-peak resolution between authentic standards of 4-deoxyorobanchol and 5-deoxystrigol. The standard mixture in a concentration of 0.5 pmol/5 µl was injected onto an Acquity UPLC® BEH C18 2.1 × 100 mm, 1.7 µm column and separated by gradient elution using 7 mM (a), 15 mM (b) and 25 mM (c) of eluent additive formic acid in the mobile phase. Figure S3. Comparison of peak areas of endogenous 4-deoxyorobanchol extracted from rice root exudate with water (blue line) or 5% acetonitrile/water (v/v, black line). Samples were purified by Oasis® HLB (Waters) and analyzed by UHPLC-ESI (+)-MS/MS using the conditions described in Table 1. The bar chart compares the compound concentration calculated in 200 ml of exudate, extracted with the two different extraction solvents. Samples were analyzed in three replicates. Error bars represent the standard deviation of the mean (± SD). Table S1. Strigolactone stability in solvents. The stability of individual analytes in water, selected organic solvents and their aqueous solutions at 0 °C during 3 (a) and 12 h (b). The results are represented in four categories of recovery range: - (0-10%), + (10-50%), ++ (50-80%), +++ (80-100%). Values are mean ± SD (n = 3). Table S2. Extraction recoveries (%) of selec [file 13007_2020_669_MOESM1_ESM.pdf]

An improved strategy to analyse strigolactones in complex sample matrices using UHPLC-MS/MS

Kristýna Floková[1,2], Mahdere Shimels[3,4], Beatriz Andreo Jimenez[3,5], Nicoletta Bardaro[6], Miroslav Strnad[2], Ondřej Novák[2] and Harro J. Bouwmeester[1*]

Additional file 1

[1]Plant Hormone Biology group, Swammerdam Institute for Life Sciences, University of Amsterdam, Science Park 904, 1098 XH Amsterdam, The Netherlands

[2]Laboratory of Growth Regulators, Institute of Experimental Botany, The Czech Academy of Sciences, and Faculty of Science, Palacký University, Šlechtitelů 27, 783 71 Olomouc, Czech Republic

[3]Laboratory of Plant Physiology, Wageningen University, Droevendaalsesteeg 1, 6708 PB Wageningen, The Netherlands

[4]Department of Microbial Ecology, Netherlands Institute of Ecology, Droevendaalsesteeg 10, 6708 PB Wageningen, The Netherlands

[5]Biointeractions and Plant Health, Wageningen University, Droevendaalsesteeg 1, 6708 PB Wageningen, The Netherlands

[6]Department of Plant, Soil and Food Science, Section of Genetics and Plant Breeding, University of Bari, via Amendola 165/A, 70126 Bari, Italy

*For correspondence, e-mail H.J.Bouwmeester@uva.nl, Tel. +31 20 5256476

Plant hormone biology, Swammerdam Institute for Life Sciences, University of Amsterdam, P.O. Box 1210, 1000 BE Amsterdam, The Netherlands

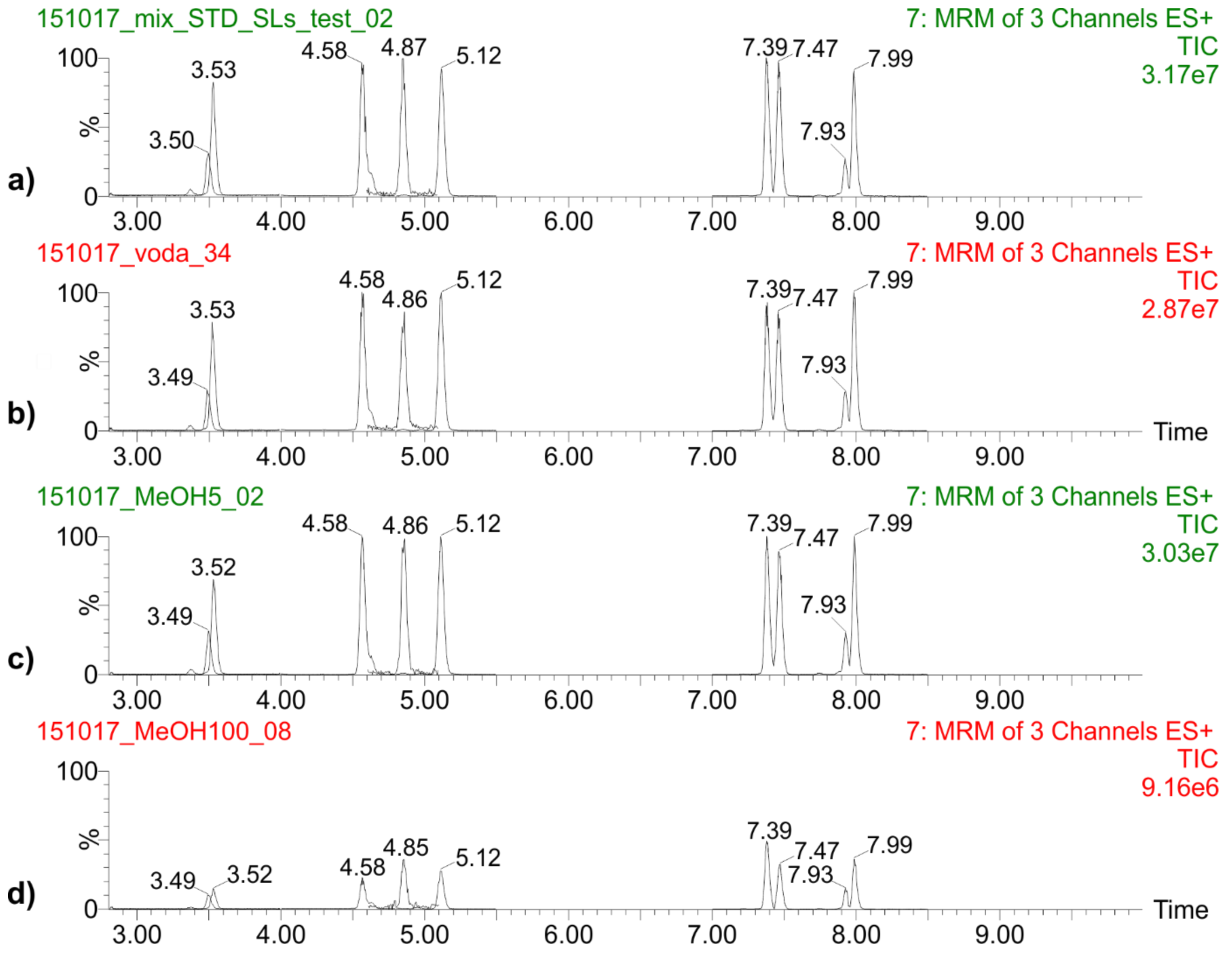

Figure S1. The comparison of peak intensity between representative chromatograms of authentic SL standards, reconstituted in 20% acetonitrile/water and injected at 0.5 pmol/5 µl at time 0 (a); and standards, incubated for 3 h in pure water (b), 5% methanol/water (v/v, c) and 100% methanol (d). Compounds eluted from the Acquity UPLC® BEH C18 2.1 × 100 mm, 1.7µm column at the following retention times (min): strigol (3.50 ± 0.01), solanacol (3.53 ± 0.01), orobanchol (4.58), sorgomol (4.86 ± 0.01), GR24 (5.12), sorgolactone (7.47; the standard mixture contains the non-natural isomer of sorgolactone, which eluted at 7.39), 4-deoxyorobanchol (7.93) and 5-deoxystrigol (7.99).

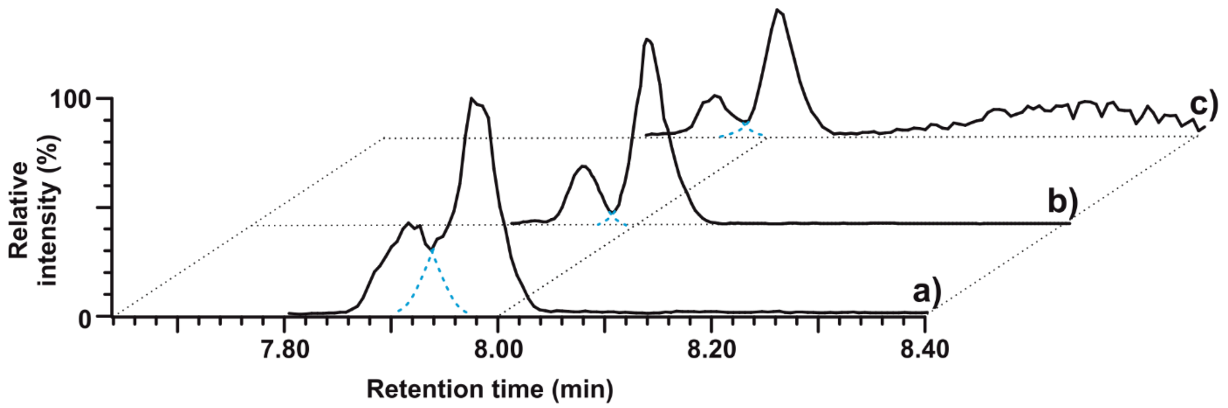

Figure S2. Comparison of peak intensity and peak-to-peak resolution between authentic standards of 4-deoxyorobanchol and 5-deoxystrigol. The standard mixture in a concentration of 0.5 pmol/5 µl was injected onto an Acquity UPLC® BEH C18 2.1 × 100 mm, 1.7µm column and separated by gradient elution using 7 mM (a), 15 mM (b) and 25 mM (c) of eluent additive formic acid in the mobile phase.

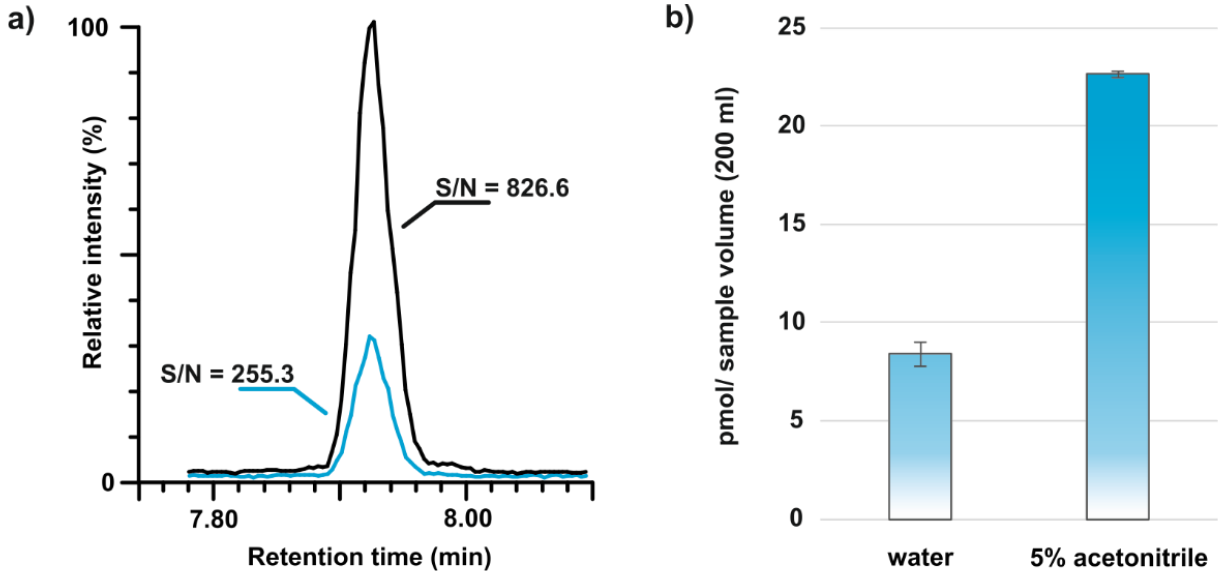

Figure S3. Comparison of peak areas of endogenous 4-deoxyorobanchol extracted from rice root exudate with water (blue line) or 5% acetonitrile/water (v/v, black line). Samples were purified by Oasis® HLB (Waters) and analyzed by UHPLC-ESI (+)-MS/MS using the conditions described in Table 1. The bar chart compares the compound concentration calculated in 200 ml of exudate, extracted with the two different extraction solvents. Samples were analyzed in three replicates. Error bars represent the standard deviation of the mean (±SD).

Table S1. **Strigolactone stability in solvents**

a) 3 h incubation		strigol	solanacol	orobanchol	sorgomol	GR24	sorgolactone	4-DO	5-DS	Average recovery (%)
Methanol	5%	+++	+++	+++	+++	+++	+++	+++	+++	100 ± 3
	10%	+++	+++	+++	+++	+++	+++	+++	+++	99 ± 2
	80%	+++	+++	+++	+++	+++	+++	+++	+++	99 ± 3
	100%	+	+	+	+	+	+	+	+	31 ± 11
Acetonitrile	5%	+++	+++	+++	+++	+++	+++	+++	+++	96 ± 4
	10%	+++	+++	+++	+++	+++	+++	+++	+++	99 ± 3
	80%	+++	+++	+++	+++	+++	+++	+++	+++	102 ± 2
	100%	+++	+++	+++	+++	+++	+++	+++	+++	102 ± 4
Acetone	5%	+++	+++	+++	+++	+++	+++	+++	+++	100 ± 3
	10%	+++	+++	+++	+++	+++	+++	+++	+++	101 ± 3
	80%	+++	+++	+++	+++	+++	+++	+++	+++	101.8 ± 3
	100%	+++	+++	+++	+++	+++	+++	+++	+++	101.4 ± 3
Water		+++	+++	+++	+++	+++	+++	+++	+++	99.2 ± 3
**b) 12 h incubation**		**strigol**	**solanacol**	**orobanchol**	**sorgomol**	**GR24**	**sorgolactone**	**4-DO**	**5-DS**	**Average recovery (%)**
Methanol	5%	+++	+++	+++	+++	+++	+++	+++	+++	97.6 ± 3
	10%	+++	+++	+++	+++	+++	+++	+++	+++	97.5 ± 3
	80%	+++	+++	+++	+++	+++	+++	+++	+++	97.2 ± 2
	100%	+	-	-	+	+	+	+	+	14.5 ± 8
Acetonitrile	5%	+++	+++	+++	+++	+++	+++	+++	+++	98.8 ± 2
	10%	+++	+++	+++	+++	+++	+++	+++	+++	97.9 ± 2
	80%	++	+++	+++	+++	+++	+++	+++	+++	95.7 ± 5
	100%	++	+	++	++	++	++	++	++	64.6 ± 15
Acetone	5%	+++	+++	+++	+++	+++	+++	+++	+++	100.2 ± 2
	10%	+++	+++	+++	+++	+++	+++	+++	+++	100.8 ± 2
	80%	+++	+++	+++	+++	+++	+++	+++	+++	96.7 ± 3
	100%	+++	+++	+++	+++	+++	+++	+++	+++	98.5 ± 4
Water		+++	+++	+++	+++	+++	+++	+++	+++	97.9 ± 2

The stability of individual analytes in water, selected organic solvents and their aqueous solutions at 0°C during 3 (a) and 12 hours (b). The results are represented in four categories of recovery range: - (0-10%), + (10-50%), ++ (50-80%), +++ (80-100%). Values are means ± SD (n = 3).

Table S2. Extraction recoveries (%) of selected strigolactones after pre-concentration with different solid-phase materials

	**Strata-X**		**Oasis HLB**		**Strata-CN**		**C18**	
C**ompound**	*Control*	*Matrix*	*Control*	*Matrix*	*Control*	*Matrix*	*Control*	*Matrix*
solanacol	97 ± 4	54 ± 35	88 ± 3	86 ± 2	96 ± 8	75 ± 1	76 ± 1	74 ± 0.3
GR24	91 ± 13	62 ± 16	98 ± 2	87 ± 1	99 ± 7	64 ± 0.01	91 ± 3	92 ± 4
[$^{2}H_{6}$]-5-DS	90 ± 1	65 ± 10	96 ± 8	95 ± 1	75 ± 1	22 ± 2	65 ± 0.1	84 ± 6

The standard mixture containing 5 pmol of solanacol, GR24 and [$^{2}H_{6}$]-5-deoxystrigol was loaded onto solid-phase materials in presence of sorghum root exudate (matrix) and matrix-free extraction solvent (control). The recovery (%) of added compounds was analyzed in the eluent. Values are means ± SD (n=3).

Table S3. Method validation

Control - Root exudates

Compound	Determined spiked content (pmol)[a]	Method precision (% RSD)[a]	Method accuracy (% bias)[a]	Determined spiked content (pmol)[b]	Method precision (% RSD)[b]	Method accuracy (% bias)[b]
strigol	1.07 ± 0.03	2.9	6.53	9.95 ± 0.09	0.9	-0.52
solanacol	0.97 ± 0.02	2.2	-3.27	9.42 ± 0.16	1.7	-5.82
orobanchol	0.96 ± 0.02	2.3	-3.53	9.55 ± 0.18	1.9	-4.53
sorgomol	1.00 ± 0.03	3.1	0.29	9.23 ± 0.06	0.7	-7.74
sorgolactone	1.00 ± 0.03	3.1	0.29	9.23 ± 0.06	0.7	-7.74
4-DO	1.07 ± 0.04	3.4	6.92	9.69 ± 0.18	1.9	-3.13
5-DS	0.98 ± 0.01	1.5	-2.28	9.75 ± 0.08	0.8	-2.48

Control - Root tissue

Compound	Determined spiked content (pmol)[a]	Method precision (% RSD)[a]	Method accuracy (% bias)[a]	Determined spiked content (pmol)[b]	Method precision (% RSD)[b]	Method accuracy (% bias)[b]
strigol	1.09 ± 0.05	4.8	0.90	9.27 ± 0.23	2.5	-7.32
solanacol	0.92 ± 0.02	2.6	-0.81	6.73 ± 0.26	3.9	-32.72
orobanchol	1.04 ± 0.02	2.3	0.42	9.22 ± 0.16	1.8	-7.81
sorgomol	1.09 ± 0.05	4.2	0.92	9.71 ± 0.18	1.9	-2.92
sorgolactone	1.05 ± 0.05	4.8	0.48	9.52 ± 0.18	1.9	-4.83
4-DO	1.03 ± 0.04	4.0	0.31	9.80 ± 0.11	1.2	-1.99
5-DS	1.05 ± 0.01	0.7	0.51	9.85 ± 0.15	1.5	-1.54

The analytical precision and accuracy, evaluated by spiking root exudate- and root tissue-free control extraction solvents with analytes at two different concentrations (1 pmol – a, and 10 pmol – b) prior to extraction. Values are means ± SD (n=4).
